# Guinea Worm (*Dracunculus medinensis*) Infection in a Wild-Caught Frog, Chad

**DOI:** 10.3201/eid2211.161332

**Published:** 2016-11

**Authors:** Mark L. Eberhard, Christopher A. Cleveland, Hubert Zirimwabagabo, Michael J. Yabsley, Philippe Tchindebet Ouakou, Ernesto Ruiz-Tiben

**Affiliations:** Centers for Disease Control and Prevention, Atlanta, Georgia, USA (M.L. Eberhard);; University of Georgia, Athens, Georgia, USA (C.A. Cleveland, M.J. Yabsley);; The Carter Center, Atlanta (H. Zirimwabagabo, E. Ruiz-Tiben);; Ministry of Public Health, N’Djamena, Chad (P.T. Ouakou)

**Keywords:** Dracunculus medinensis, Phrynobatrachus francisci, wild frog, Guinea worm, Chad, paratenic host, Guinea worm disease, dracunculiasis, infective larvae, transmission, parasites, humans, dogs, zoonoses

## Abstract

A third-stage (infective) larva of *Dracunculus medinensis*, the causative agent of Guinea worm disease, was recovered from a wild-caught *Phrynobatrachus francisci* frog in Chad. Although green frogs (*Lithobates clamitans*) have been experimentally infected with *D. medinensis* worms, our findings prove that frogs can serve as natural paratenic hosts.

The peculiar epidemiology of *Dracunculus medinensis* (Guinea worm), the causative agent of dracunculiasis (Guinea worm disease), in Chad has led to speculation that a paratenic host is involved in the life cycle, most likely an animal with an aquatic stage that would feed upon copepods and harbor the infection for subsequent transmission to a human or dog definitive host (*1*). Recent experiments demonstrated that *D. medinensis* worms, like the closely related parasite *D. insignis*, could utilize green frog (*Lithobates clamitans*) tadpoles as a paratenic host (*2*). During June and July 2016, a survey of potential *D. medinensis* worm paratenic hosts was conducted in Chad. The study area was located in southern Chad near the small village of Marabe (Moyen Chari region, Kyabe district), along the upper reaches of the Chari River, where many infections in dogs have been recorded; the closest large town to Marabe is Sarh (*3*).

## The Study

We used standard procedures, as previously described (*1*), to examine muscle and viscera of 88 frogs from the study area; the frogs, which were of several sizes and species (i.e., Ranidae, Pipidae, Phrynobatrachidae, Bufonidae), were collected by local villagers and fishermen. In brief, the viscera was removed and placed in water for at least 1 h before being examined by microscope for motile nematode larvae. The musculature and carcass were bluntly dissected and similarly placed in water for at least 1 h before the solution was examined for motile nematode larvae.

We observed 1–5 nematode larvae in 6 (7%) of the 88 frogs. Morphologically similar larvae were collected from the viscera washing of 5 of the 6 frogs; these larvae were identified as pinworms, based on morphologic characteristics and comparison to larvae released by a female oxyurid collected from the gut. However, upon subsequent microscope examination, 1 larva from the muscle and carcass washings of a single mature frog was found to be morphologically consistent with *Dracunculus* species, including size, distinct cuticular striation, and, most notably, a 3-lobed tail (Figure). We preserved the larva in ethanol and then extracted DNA and amplified a partial cytochrome c oxidase subunit I gene by PCR (*4*). Partial sequencing (187 bp) showed that the larva shared 99.5% similarity with *D. medinensis* isolates in GenBank (accession nos. LK978189 and KF770021– KF770024), confirming its identity as *D. medinensis*. The sequence shared only 95.2% similarity with *D. insignis* and 91.9% similarity with *D. lutrae* (GenBank accession nos. EU646534 and EU646602, respectively). 

**Figure:**
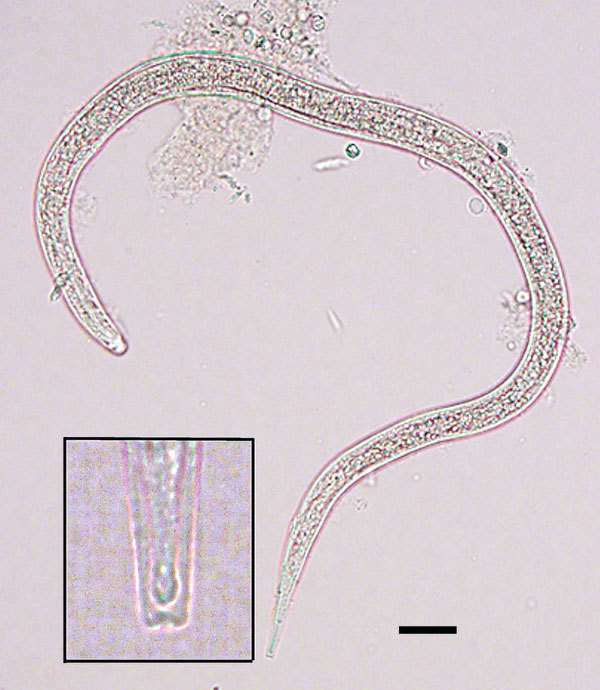
Size and shape of a *Dracunculus medinensis* third-stage larva recovered from a *Phrynobatrachus francisci* frog from Chad. Scale bar indicates 25 μm. Inset shows detailed morphology of the tip of the tail of the larva, including the characteristic 3-lobed tip.

To confirm the species identity of the frog, we extracted DNA from ethanol-fixed tissue and amplified the 16S ribosomal RNA gene (*2*). The sequence (450 bp) indicated that the frog was a ranid species in the genus *Phrynobatrachus*, most likely *P. francisci* because it shared 99% similarity with *P. francisci* sequences in GenBank (accession nos. GU457546–GU457549, EU71820, and AY902377).

## Conclusions

Tadpoles and frogs have long been known to experimentally support infective larvae of *D. insignis* (*5*–*7*), and just recently, they have been shown to experimentally support infective larvae of *D. medinensis* (*2*); however, natural infection with *Dracunculus* species has not previously been documented in any wild-caught amphibian. The finding of a wild-caught frog harboring a natural infection with a *D. medinensis* larva validates the findings of these experimental infections and demonstrates that such a paratenic host is likely involved in the transmission of *D. medinensis* larvae in Chad. This finding is especially noteworthy at this point because the Guinea worm eradication program has reduced the number of countries with endemic Guinea worm disease from 20 to 4 and the number of persons infected each year from >3 million in 1986 to <20. To be uncovering this aspect of the *D. medinensis* life cycle, the description of which was published >145 years ago (*8*) and remained relatively unchanged to date, further highlights the need to continue field research, even at the end of an eradication campaign.

Given the diversity of frog species (i.e., families Ranidae, Pipidae, and Phrynobatrachidae) that can be infected with *D. medinensis* or *D. insignis* worms, it seems probable that natural *Dracunculus* infections are not limited to frogs of the genus *Phrynobatrachus* but may well include numerous other ranids and highly aquatic *Xenopus* species frogs (African clawed frogs), which are common and native to Chad. Additional surveillance is needed to detail the prevalence and burden of infection among frogs in Chad as well as the diversity of natural hosts. These data do not address whether all transmission occurring in humans and dogs in Chad are a result of consumption of a paratenic host, such as a frog, but the peculiar epidemiology of *D. medinensis* worms in Chad clearly suggests that traditional drinking water sources are not the primary source of infection. 

Our findings confirm that an appropriate wild-caught paratenic host in Chad was infected with a *D. medinensis* larva, and they corroborate findings of experimental studies that suggested the possible inclusion of an amphibian paratenic host in the maintenance of *D. medinensis* worms in nature. We conclude that paratenic hosts, specifically frogs, may facilitate transmission of *D. medinensis* worms to humans and dogs in Chad via consumption of poorly cooked or raw food items.
